# Functional Connectivity Reveals Which Language the “Control Regions” Control during Bilingual Production

**DOI:** 10.3389/fnhum.2016.00616

**Published:** 2016-11-30

**Authors:** Le Li, Karen Emmorey, Xiaoxia Feng, Chunming Lu, Guosheng Ding

**Affiliations:** ^1^State Key Laboratory of Cognitive Neuroscience and Learning and IDG/McGovern Institute for Brain Research, Beijing Normal UniversityBeijing, China; ^2^Laboratory for Language and Cognitive Neuroscience, School of Speech, Language, and Hearing Sciences, San Diego State UniversitySan Diego, CA, USA; ^3^Centre for Collaboration and Innovation in Brain and Learning SciencesBeijing, China

**Keywords:** language control, functional connectivity, dorsal anterior cingulate cortex, left caudate nucleus, bimodal bilinguals, signed language

## Abstract

Bilingual studies have revealed critical roles for the dorsal anterior cingulate cortex (dACC) and the left caudate nucleus (Lcaudate) in controlling language processing, but how these regions manage activation of a bilingual’s two languages remains an open question. We addressed this question by identifying the functional connectivity (FC) of these control regions during a picture-naming task by bimodal bilinguals who were fluent in both a spoken and a signed language. To quantify language control processes, we measured the FC of the dACC and Lcaudate with a region specific to each language modality: left superior temporal gyrus (LSTG) for speech and left pre/postcentral gyrus (LPCG) for sign. Picture-naming occurred in either a single- or dual-language context. The results showed that in a single-language context, the dACC exhibited increased FC with the target language region, but not with the non-target language region. During the dual-language context when both languages were alternately the target language, the dACC showed strong FC to the LPCG, the region specific to the less proficient (signed) language. By contrast, the Lcaudate revealed a strong connectivity to the LPCG in the single-language context and to the LSTG (the region specific to spoken language) in the dual-language context. Our findings suggest that the dACC monitors and supports the processing of the target language, and that the Lcaudate controls the selection of the less accessible language. The results support the hypothesis that language control processes adapt to task demands that vary due to different interactional contexts.

## Introduction

How bilinguals control their languages is a major focus of research because language processing places clear demands on cognitive control processes for bilingual speakers (e.g., Kroll et al., 2014). The engagement of cognitive control processes is believed to be due to conflicts arising from co-activation of the non-target language (Colomé, 2001; Kroll et al., 2006; Wu and Thierry, 2012) or due to less exposure to and less experience speaking each language compared to monolingual speakers (Michael and Gollan, 2005; Gollan et al., 2008; Costa and Sebastián-Gallés, 2014). Language conflicts occur not only when both languages are used alternately (e.g., during language switching), but also when only one language is used within an entire experimental session (Wu and Thierry, 2010; Green, 2011). Language control appears to be necessary in language-switching contexts as well as in contexts where bilinguals only need to use one of their languages.

Neuroimaging studies have shown that several brain regions are engaged in bilingual language control, including the prefrontal cortex, the dorsal anterior cingulate cortex (dACC) and subcortical structures (Abutalebi and Green, 2007; Green and Abutalebi, 2013). Among these regions, the dACC and the left caudate nucleus (Lcaudate) have been most frequently reported to be engaged during language control (Crinion et al., 2006; Abutalebi et al., 2012; Zou et al., 2012b; Li et al., 2015; Branzi et al., 2016). The function of the dACC is generally associated with monitoring speech production, but precisely what processes are monitored is under dispute (Luk et al., 2012). On one hand, the dACC has been found to be strongly activated in situations with a high degree of language conflict, such as in a language-switching task, which suggests a role for the dACC in monitoring the conflict that arises from activation of words in the non-target language (Abutalebi et al., 2008; van Heuven et al., 2008). On the other hand, language production in monolinguals can also activate the dACC, indicating that its function is not exclusive to bilingual language control (Price, 2012; Abutalebi et al., 2013). Some researchers have thus identified the role of the dACC in speaking as initiating speech production in general (Luk et al., 2012; Price, 2012). In this regard the dACC is assumed to control the target language in bilinguals, rather than in monitoring conflict from the non-target language. The dispute between these views is primarily concerned with which language the dACC exerts control over during speaking.

As for the Lcaudate, previous research has suggested that it plays a role in language selection, which is particularly critical for controlling the less proficient language (Tan et al., 2011; Zou et al., 2012b). In a functional MRI study with children, Tan et al. (2011) found that the activity of the Lcaudate in a reading task could predict later reading performance in the second language (L2) but not in the native language (L1). In another study, the Lcaudate in trilingual speakers was found to be engaged to a greater extent for the less proficient language in a production task (Abutalebi et al., 2013). However, Aglioti et al. (1996) reported a case study in which a lesion to the Lcaudate led to a dysfunction in language selection which was more prominent for the L1, rather than for the L2. Additionally, some studies report equal impairment in the two languages for bilingual aphasic patients with damage to the Lcaudate, suggesting it plays a role in the selection of both languages (Abutalebi et al., 2000; Marien et al., 2005). Thus, the findings from lesion studies do not seem to be completely in accord with the findings from neuroimaging studies. It is still to be determined whether the Lcaudate mainly exerts control on the less proficient language during speaking or is generally involved in the selection of both languages.

Notably, how these control regions interact with the language system may depend on the context of language use. Green and Abutalebi (2013) have proposed the adaptive control hypothesis which states that control processes in bilinguals will change depending upon the nature of the interactional context. They classified three types of interactional contexts: a single-language context (one language is used in one session and the other language is used in a different session), a dual-language context (both languages are used and switched frequently within a session but not within an utterance), and a dense code-switching context (languages are interleaved in the course of an utterance). Different interactional contexts involve different control demands. For example, task engagement and selective inhibition processes are required in a dual-language context, but not in a single-language context. Previous research on bilingual language production compared naming latencies between dual- and single-language contexts for L1 and L2, and the results showed that the context effect (i.e., slower reaction times in the dual-language context) was greater for the L1 than the L2 (Christoffels et al., 2007). This asymmetrical effect is argued to be a consequence of the balance of activation levels for the two languages in the dual-language context (De Groot and Christoffels, 2006). Therefore, control processes are engaged differently across these interactional contexts and different control regions are recruited. Interactional contexts could also explain the diversity of previous neuroimaging and lesion-based results regarding the role of the dACC and Lcaudate in language control.

The present study aims to clarify the control functions of the dACC and the Lcaudate in bilingual language production by investigating single- and dual-language contexts. We did not examine a dense code-switching context because in this interactional context code-switching occurs within an utterance (Heredia and Altarriba, 2001; Green and Abutalebi, 2013), and our study only examined single word production. To investigate the function of the dACC and Lcaudate, we considered not only neural activation within these regions, but also their functional connectivity (FC) with other brain areas. FC is generally defined as the temporal correlation between time-series in two brain regions (Friston, 1994). In a previous study, we examined the FC of the dACC with language regions in bimodal bilinguals whose L1 was Mandarin and whose L2 was Chinese Sign Language (CSL), in comparison to monolingual Mandarin speakers (Li et al., 2015). The results showed that during spoken language production, the FC of dACC and a brain region associated with speech production (the left middle superior temporal gyrus, LSTG) was stronger for bimodal bilinguals than for monolinguals. We hypothesized that the stronger FC for the bilinguals reflected greater control demands when speaking their L1 because this language was relatively less practiced compared to the monolinguals who only spoke Mandarin (Michael and Gollan, 2005; Bialystok, 2009). We further hypothesized that the dACC may primarily exert control over the target language, given that the bilinguals did not display an increase of FC between the dACC and a region associated with the non-target (signed) language (i.e., the left superior pre/postcentral gyrus, LPCG). However, in that study we only investigated the FC in an L1 naming task. A stringent test of our hypothesis requires examination of the FC across L1 production and L2 production and across single- and dual-language contexts.

In the current study, we manipulated these variables to clarify how the dACC and the Lcaudate control each language during word and sign production. Crucially, we tested bimodal bilinguals who use both a spoken and a signed language. Bimodal bilinguals provide a special perspective for exploring these issues because their two languages are in different modalities which depend on partially distinct neural systems (MacSweeney et al., 2008; Emmorey and McCullough, 2009; Korzeniewska et al., 2011; Zou et al., 2012a; Emmorey et al., 2014). Thus, the neural substrates underlying the processing of each language can be distinguished to some extent. In contrast, for unimodal bilinguals who speak two aural-oral languages, both of their languages engage the same set of brain regions (e.g., Chee et al., 2003; Perani and Abutalebi, 2005; Simos, 2005), and thus it is very difficult to separate the two languages of unimodal bilinguals within the brain. However, it is important to note that signed and spoken languages conform to the same universal linguistic properties and principles (e.g., Sandler and Lillo-Martin, 2006), and in this respect bimodal bilinguals are comparable to unimodal bilinguals.

We carried out two functional MRI experiments^1^. In the first experiment, blocked picture naming tasks were conducted in a single-language context where only one language was used to name the presented pictures. The tasks were completed in L1 (spoken language) and L2 (signed language), respectively. We calculated the FC between the targeted control regions (dACC and Lcaudate) and a brain region that was specific to each language, and we compared the FC between the naming tasks in L1 and L2. For signed language, we selected the left superior pre/postcentral gyrus (LPCG) as the target brain region, and for spoken language, we chose the left middle superior temporal gyrus (LSTG). For hearing signers, the LPCG always shows strong activation during signed language production, but not during spoken language production (Braun et al., 2001; Emmorey et al., 2007, 2014; Zou et al., 2012a). We chose the LSTG as the target brain region for spoken language because this region is more strongly activated when speaking compared to signing (Zou et al., 2012a; Emmorey et al., 2014). Although signed language processing has been reported to activate posterior perisylvian regions, the region most associated with sign production is located at a more posterior portion of the superior temporal cortex (Petitto et al., 2000; MacSweeney et al., 2002; Emmorey et al., 2014). In contrast, the LSTG region selected here for spoken language is located at the middle portion of superior temporal cortex (anterior to the primary auditory cortex).

In the second experiment, two picture naming tasks were performed in a dual-language context, which included a “fixed-switch” and a “random-switch” picture-naming task. In the fixed-switch task, the signed language and the spoken language were alternately used to name the pictures, from trial to trial. In the random-switch task, the signed language and the spoken language were randomly used to name the pictures, according to the cue displayed after picture presentation. Notably, the fixed-switch task and the random-switch task created a dual-language context rather than a dense code-switching context as defined by Green and Abutalebi (2013). In both tasks the nouns produced based on the pictures could not be combined to form an utterance. So the language switching in these tasks was considered not within an utterance. More importantly, the output language is determined by the cue rather than opportunistically planned by the speakers. Thus for both tasks the two languages were in a competitive relationship, and were not in a co-operative relationship which is a unique characteristic for the dense code-switching context (Green and Abutalebi, 2013). We compared the FC of the control regions in the fixed-switch task to that in the single-language tasks from Experiment 1. In order to better understand the function of both control regions in the dual-language context, we further measured the regional activation of dACC and Lcaudate when processing each language within the random-switch picture-naming task.

## Experiment 1

### Methods

#### Participants

A group of 14 bimodal bilinguals (4 males; mean age = 49.5) took part in the first experiment. All participants were teachers in bilingual deaf schools, and taught deaf children using CSL every day. They were native speakers of Mandarin and acquired CSL as their L2 later in life (mean age = 21). They had been signing for at least 12 years (mean = 28.5 years), and used CSL frequently in daily life. They self-rated their CSL as very proficient (mean = 4.5 on a scale of 1–5, where a larger value means more proficient), as well as their Mandarin (mean = 5). None of the participants had a history of neurological disease, inpatient psychiatric care or head injury, and all of them were right handed according to the Edinburgh Handedness Inventory (Oldfield, 1971). Before the experiments, informed consent was obtained from all participants. The present study was approved by the Institutional Review Board of Beijing Normal University Imaging Center for Brain Research.

#### Stimuli and Task Design

Eighty black-and-white pictures (line drawings) were selected from a standard database (Zhang and Yang, 2003). We screened each picture to make sure that naming the picture in CSL or in Mandarin would incur minimal head movement. We excluded any pictures that involved movement of legs or head when naming with signs. The stimuli were divided into two sets for each of two tasks with the following matched indices for Mandarin: word length (mean = 1.80 vs. 1.98 characters, *p* = 0.174, two-tailed, the same below), naming agreement (mean = 0.93 vs. 0.97, *p* = 0.126), familiarity (mean = 4.65 vs. 4.68 on a scale of 1–5, *p* = 0.684) and imaginability (mean = 3.71 vs. 3.73 on a scale of 1–5, *p* = 0.856). The picture sets were also matched on visual complexity (mean = 2.53 vs. 2.53 on a scale of 1–5, *p* = 0.991).

**Figure 1:**
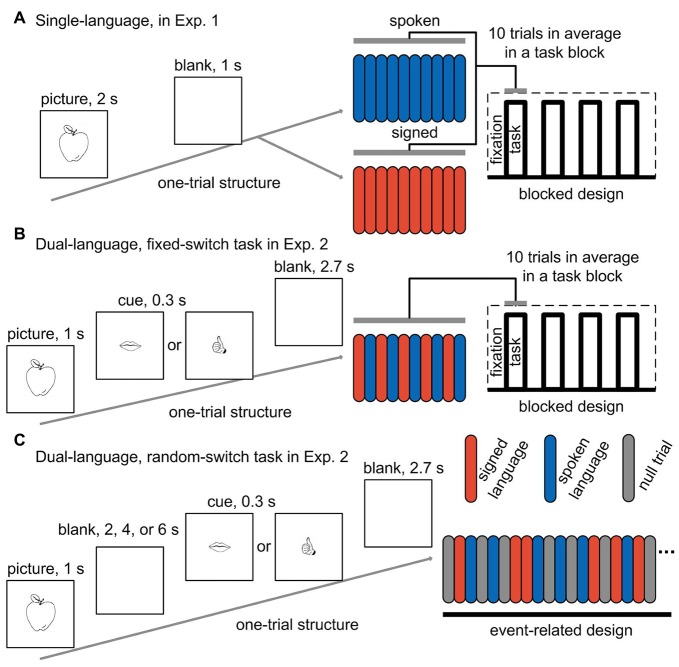
**Experimental paradigms for the tasks in both experiments.** One-trial structure, task-block structure and the blocked design are shown for the two tasks (one with spoken language and the other with signed language) in a single-language context **(A)**, and for the fixed-switch task in a dual-language context **(B)**. One-trial structure and the event-related design are shown for the random-switch task in a dual-language context **(C)**. Red bar, signed language trial; blue bar, spoken language trial; gray bar, null fixation trial; Exp. 1, Experiment 1; Exp. 2, Experiment 2.

Picture-naming was performed during brain scanning in a single-language context where only one language was used in a run. There were two successive functional scanning runs, one for each language. Blocked design was adopted, and each run consisted of four task blocks alternated with five fixation blocks. There were 40 trials across a run. In each trial, a picture was presented in the center of the screen for 2 s, followed by a 1-s blank screen (see Figure 1A for an illustration of the design). The participants were asked to name the pictures with Mandarin or CSL, and the target language was indicated before the start of a run. Half of the participants first completed a spoken language run and then a signed language run, while the other half performed the tasks in the reverse order. The picture sets were also counter-balanced across participants. Before the participants entered into the scanner, they performed 10 practice trials for the task with each language.

#### MRI Acquisition

fMRI data was collected with a 3T Siemens Trio Scanner at the MRI Center in Beijing Normal University, using T2-weighted gradient-echo echo planar imaging (EPI) sequence. The functional scanning parameters were as follows: TR = 2000 ms, TE = 20 ms, flip angle = 80°, FOV = 220 mm, slice number = 32 axis slices, slice thickness = 4.8 mm, voxel size 3.1 mm × 3.1 mm × 4.8 mm, series = interleaved. For each run, 135 functional volumes were collected. High-resolution T1-weighted anatomical images were also acquired for all participants using the MPRAGE sequence, to provide better estimates for the normalization of functional images to the MNI space. The parameters of anatomical imaging were as follows: TR = 2530 ms, TE = 3.39 ms, flip angle = 7°, FOV = 256 mm, matrix = 256 × 256, slice number = 128 sagittal slices, slice thickness = 1.33 mm, voxel size = 1. 3 mm × 1. 0 mm × 1.0 mm, series = interleaved.

#### Region of Interest Definition

**Figure 2:**
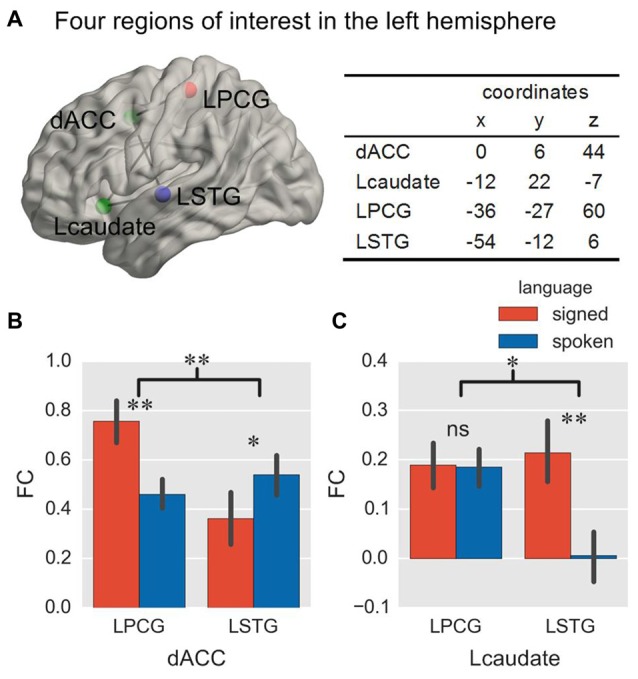
**Location of ROIs and ROI-wise FC in Experiment 1.** The location of two control ROIs (green sphere) and two language-specific ROIs (red and blue sphere) in the brain is displayed from a left lateral view, and their coordinates are shown in the table **(A)**. The histograms show the FC with the two language-specific regions in a single-language context, for the dACC **(B)** and the Lcaudate **(C)**. *p < 0.05; **p < 0.01; ns = not significant. The error bar represents the standard deviation. ROI, region of interest; FC, functional connectivity; dACC, dorsal anterior cingulate cortex; Lcaudate, left head of caudate nucleus; LPCG, left superior pre/postcentral gyrus; LSTG, left middle superior temporal gyrus.

The goal of the present study was to explore how control regions are functionally connected to the languages of bimodal bilinguals during language production by comparing their functional connections with language regions specific to sign or speech production. We selected the dACC and Lcaudate as the control regions of interest (ROIs), based on the work from previous studies (Abutalebi et al., 2012; Zou et al., 2012b), and we chose the left superior pre/postcentral gyrus (LPCG, located at the boundary of the postcentral gyrus in parietal cortex) and left middle superior temporal gyrus (LSTG) as the ROIs specific to signed language and spoken language, respectively (Emmorey et al., 2014). Other regions underlying the processing of both languages (e.g., Broca’s area) were not included because they could not differentiate between the two languages. The coordinates of the ROIs were defined from our previous study (Li et al., 2015; see Figure 2A for their locations and coordinates). A sphere was created for each ROI with a radius of 6 mm.

#### fMRI Data Preprocessing

We conducted data preprocessing with SPM 8. Specifically, functional images were first corrected for slice acquisition timing difference and head motion. Individual anatomical images were coregistered with the corresponding mean functional images. Then the functional images were normalized to the Montreal Neurological Institute (MNI) space through the obtained spatial warping parameters, and resampled to a spatial resolution of 3 mm × 3 mm × 3 mm. The normalized images were smoothed with an isotropic 6 mm full width at half maximum (FWHM) Gaussian kernel and high-pass filtered with a cut-off frequency of 1/128 Hz. For the analyses of FC, we further regressed out the covariates of six motion parameters, white matter signal and cerebrospinal fluid signal, to control for nuisance effects from head motion and physiological signal (Fox et al., 2009).

#### Functional Connectivity Analysis

To compute ROI-wise FC between the control and language regions, we first averaged the BOLD time series of all voxels within each ROI. Three frames (6 s) at the beginning of task blocks were excluded and three frames after the end of task blocks were included in the task-related time series, in order to account for hemodynamic delay (Fair et al., 2007). The BOLD signals corresponding to each task block were extracted, converted to normalized scores, and concatenated. This was done for the spoken language run and the signed language run, respectively. Then we calculated Pearson correlation of the signals between four pairs of ROIs, namely dACC—LPCG, dACC—LSTG, Lcaudate—LPCG and Lcaudate—LSTG. The correlation coefficients obtained for each participant and each language were converted to Fisher’s *Z* scores for statistical analyses.

We used SPSS 20 to conduct group-level statistical analyses. A repeated-measures ANOVA on the FC (values) was carried out for each control region (either the dACC or the Lcaudate), with the connection to language regions (LPCG and LSTG) and the language in use (Mandarin and CSL) as within-subject factors. If there was a significant interaction between connection (to language regions) and language (in use), then simple effect analyses were further performed by examining the language effect on FC for each connection with paired-sample *T*-tests. Considering the relatively small sample of participants in the present study, we further conducted bootstrap tests to evaluate the confidence level of FC difference for the above statistical analyses. Bootstrap tests can check the stability of the results and do not require a normal distribution of the data (Efron and Tibshirani, 1994). In brief, we constructed a null hypothesis for the test and created a fake population by shifting the original data by removing the mean difference between conditions. For each iteration, a bootstrap sample of observations with a size equal to the original sample was randomly selected with replacement from the fake population, and a statistical analysis on this sample was performed. This procedure was repeated 1000 times, so that a distribution of 1000 statistics under the null hypothesis was obtained. The significance level was determined by the quantile of the original test statistic in the distribution.

### Results

#### Functional Connectivity

We first explored whether the control regions (dACC or Lcaudate) interacted with the target language region (LPCG or LSTG) in the single-language context. Here we mainly contrasted the FC of each connection between the languages, and the language effect between connections (that is, the interaction effect) since it is not possible to directly contrast the FC between different regions (or connections). The ANOVA on the FC of the dACC showed a significant interaction of connection × language (Table 1 and Figure 2B; *F* = 16.72, *p* = 0.001, and *p* = 0.007 in the bootstrap test). All tests in this study were two-tailed, unless specified otherwise. There were no significant main effects for the connection factor (*F* = 2.21, *p* = 0.161; *p* = 0.179 in the bootstrap test) and for the language factor (*F* = 0.79, *p* = 0.397; *p* = 0.381 in the bootstrap test). The subsequent simple effect analysis revealed that for the connection of dACC—LPCG, this FC was stronger for signed than spoken language production (*p* = 0.009 in *T*-test and *p* = 0.013 in the bootstrap test). In contrast, the FC for the dACC—LSTG was stronger during spoken than signed language production (*p* = 0.044 in *T-test* and *p* = 0.040 in the bootstrap test). As predicted, the results indicate the dACC functionally interacts with the region for the target language, rather than the non-target language or both languages.

**Table 1:** **Region of interest (ROI)-wise FC of the control and language regions in single- and dual-language contexts**.

Connection	Single-language context			Dual-language context
	Signed language	Spoken language	Averaged	Fixed-switch task
dACC—LPCG	0.76**	0.46	0.61	0.62
dACC—LSTG	0.36	0.54*	0.45	0.28*
Lcaudate—LPCG	0.19	0.19	0.19	0.07**
Lcaudate—LSTG	0.21**	0.01	0.11	0.23*

There are four connections between pairs of ROIs. For each connection, the FC was compared between signed and spoken languages in the single-language context; and the FC in the fixed-switch task was compared to the averaged FC in the single-language context. *p < 0.05; **p < 0.01. FC, functional connectivity; dACC, dorsal anterior cingulate cortex; Lcaudate, left head of caudate nucleus; LPCG, left superior pre/postcentral gyrus; LSTG, left middle superior temporal gyrus.

In contrast, the ANOVA on the FC for the Lcaudate showed a significant interaction of connection × language (Table 1 and Figure 2C; *F* = 6.06, *p* = 0.029; *p* = 0.036 in the bootstrap test), as well as a significant main effect of language (*F* = 4.87, *p* = 0.046; *p* = 0.045 in the bootstrap test). A simple effect analysis revealed that the FC for the Lcaudate—LPCG was not different across signed and spoken languages (*p* = 0.953 in *T*-test and *p* = 0.960 in the bootstrap test), while the FC of the Lcaudate—STG was stronger in signed than spoken language production (*p* = 0.003 in *T-test* and *p* = 0.007 in the bootstrap test). These results thus suggest that the connection of Lcaudate is stronger when the less proficient signed language is produced.

## Experiment 2

### Methods

#### Participants

Another group of 14 bimodal bilinguals (3 males; mean age = 49) participated in the second experiment (seven of them had participated in Experiment 1). As in Experiment 1, all participants were teachers in bilingual deaf schools and taught deaf children with CSL every day. They were also native speakers of Mandarin and acquired CSL as their L2 later in life (mean age = 19). They self-rated both of their languages as very proficient (mean = 5 for Mandarin and 4.35 for CSL on a scale of 1–5). They had been signing for a minimum of 15 years (mean = 30 years). All participants were right handed according to the Edinburgh Handedness Inventory. None reported a history of neurological disease, inpatient psychiatric care, or head injury. Informed consent was obtained from all participants before the experiment.

#### Stimuli and Task Design

To examine how the dACC and Lcaudate controlled the language system in a dual-language context, two language-switching tasks were used: a fixed-switch picture-naming task that we used to calculate FC of the control regions, and a random-switch task that we used to compute regional activation of the control regions (see below). Eighty-two black-and-white line-drawn pictures were selected from the same standard database as in Experiment 1 (Zhang and Yang, 2003). Forty pictures were used for the fixed-switch task, and the remaining 42 were used in the random-switch task. There were two scanning runs for each task. The stimuli were presented once in each scanning run for each task.

During the fixed-switch task, CSL and Mandarin were used alternately from trial to trial. The fixed-switch task involved a blocked design that was comparable to the design for the task in Experiment 1. In each run, four task blocks alternated with five fixation blocks, and each run contained 40 trials in total. Since the language-switching task was more difficult than the picture-naming task in a single-language context, the duration of a trial was set to 4 s, which was 1 s longer than that for the tasks in Experiment 1. In each trial, the picture was presented for 1 s, followed by a cue for 0.3 s reminding participants of which language to use in this trial (the cue was a drawing of either a mouth or a hand denoting spoken or signed language, respectively). After the cue, a blank screen was presented for 2.7 s (see Figure 1B for an illustration of the paradigm). The participants were asked to name the pictures in Mandarin and CSL by turns.

Ideally, we would like to calculate the activation of the control regions when producing each language to examine whether these regions are differentially engaged for each language in a dual-language (switching) context. However, since participants rapidly produced the two languages in the fixed-switch task, the functional activation could not be separated in the temporal domain for each language. Thus, we designed a random-switch picture-naming task using a rapid event-related design in order to compare the activation of the control regions when producing each language in a dual-language context.

The random-switch task was performed in a separate set of two scanning runs. We applied a delayed naming design, in which the cue indicating which language to use was displayed several seconds after picture presentation (see Figure 1C for an illustration of the paradigm). With this design, we could eliminate the activation derived from picture processing and concept access. In each trial, a picture was presented for 1 s, and then a blank screen appeared for an average of 4 s (jittered from 2 s to 6 s). After the blank screen, a cue was displayed for 0.3 s, followed by another blank screen for 2.7 s. The participants were asked to name the picture in either CSL or Mandarin according to the cue (i.e., a drawing of either a mouth or a hand). The spoken- and signed-language cues were presented in an unpredictable pseudorandom sequence, so that the number of switch and non-switch trials was equal, and the number of consecutive trials using the same language was not more than four. In each run, there were 20 trials for spoken Mandarin and 22 trials for CSL. Twenty-five null events of fixation were also added into a run. The sequence of events in a run was optimized to separate the activation of picture vs. cue, and spoken vs. signed language during cue phase.

#### Behavioral Measures

Because a language response recording device was unavailable in the scanner, we recorded the reaction times for language production outside scanner after scanning in a separate session for ten participants (four participants were not able to participate in this session due to time limitations). Behavioral data were collected for the two language conditions for the fixed-switch task only. In addition, for the purpose of comparing reaction times between single- and dual-language contexts, the participants were asked to name pictures in a single-language block (either CSL or Mandarin)—the same task that was used in Experiment 1. We did not collect the behavioral data for the random-switch task since it was not comparable to the single-language production task due to the delayed naming design. Thus, there were four conditions for which we have behavioral naming data: dual-spoken condition, dual-signed condition, single-spoken condition and single-signed condition. The mixing cost for each language was computed as the difference in reaction times for that language between the dual- and single-language conditions. Note that the mixing cost here is different from a switch cost, which is generally defined as the difference in reaction times between switch and non-switch trials in a dual-language context. A 2-by-2 repeated-measures ANOVA on reaction times was conducted to examine the difference in mixing cost across the two languages.

#### MRI Acquisition

The scanning data for Experiment 2 was also collected at the MRI Center in Beijing Normal University, with the same acquisition sequences and parameters as in Experiment 1 for the functional and structural scanning. One-hundred and forty-one functional images were acquired for each run in the fixed-switch task, while 166 were obtained for each run in the random-switch task.

#### Region of Interest Definition

The ROIs were the same as those in Experiment 1: dACC, Lcaudate, LPCG and LSTG.

#### fMRI Data Preprocessing

The same as in Experiment 1.

#### Functional Connectivity Analysis

We investigated the FC between control regions (dACC, Lcaudate) and language regions (LPCG, LSTG) in the fixed-switch task. We first computed for each connection the mean FC (correlation coefficient *Z* score) averaged across the single-language production tasks for the two languages and across all participants in Experiment 1. The obtained averaged FC served as an expected value for the FC for the fixed-switch task. If a control region is connected by turns with each target language when the languages are switched, then the observed FC with the regions for the two languages in the fixed-switch task should be approximately equal to the expected value from single-language production (derived from Experiment 1). Alternatively, if a control region is connected with only one of the two languages in the fixed-switch task, then the observed FC should be more prominent for the region specific to that language than the other language. The observed ROI-wise FC for each participant in the fixed-switch task was analyzed by correlating the task-related time series between the ROIs. The task-related time series were extracted in the same way as in Experiment 1, except that the normalized time series from the two runs of the fixed-switch task were merged. After that, we examined whether the observed FC was different from its expected value with one-sample *T-tests*, and we examined whether the difference for the observed FC, relative to its expected value, was similar for the two language-specific regions (LPCG vs. LSTG) using paired-sample *T*-tests.

#### Brain Activation Analysis

The activation of the control regions was compared between languages in the random-switch task, in order to provide more evidence for how the dACC and Lcaudate control each language in a dual-language context. To compute individual brain activation, we built a general linear model into which the four regressors of events were entered, including picture, spoken language, signed language and fixation. Each regressor was modeled by convolving a delta function time-locked to each event with the hemodynamic response function. Then we extracted and averaged the activation from voxels across each ROI for each language. At the group-level analysis, differences in activation between languages were examined for the dACC and Lcaudate with paired-sample *T*-tests.

### Results

#### Behavior Outside the Scanner

**Figure 3:**
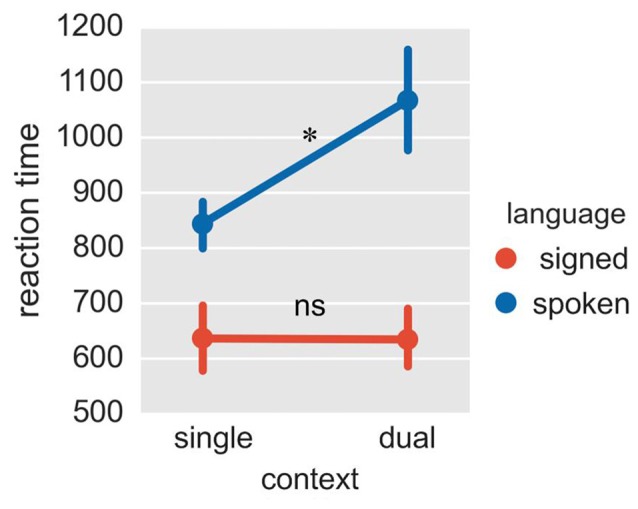
**Reaction times in Experiment 2.** The plot shows reaction times in four conditions of context × language. The reaction times were collected for signed and spoken languages, both in the fixed-switch naming task and in the single-language naming tasks. *p < 0.05; ns = not significant. The error bar represents the standard deviation.

Given that spoken and signed languages are in different modalities, the response latency for these two languages is not directly comparable. Therefore, we mainly focused on whether the mixing cost (reaction times in the fixed-switch task minus reaction times in the single-language task for that language) differed between Mandarin and CSL. The ANOVA on reaction times revealed a significant interaction between language and task (Figure 3; *F* = 9.09, *p* = 0.015; *p* = 0.036 in the bootstrap test). The mixing cost was significant for Mandarin (cost = 223 ms, *p* = 0.036 in *T*-test; *p* = 0.076 in the bootstrap test), but not for CSL (*p* = 0.971 in *T-test*; *p* = 0.975 in the bootstrap test). Although the mixing cost was marginally significant for Mandarin in the bootstrap test, it was significant in a one-tailed test (*p* = 0.038). A one-tailed test is appropriate because the difference between conditions is expected to be positive (i.e., a mixing cost is expected). The greater mixing cost for the L1 (Mandarin) is consistent with previous findings with unimodal bilinguals (Christoffels et al., 2007). This pattern may indicate that L1 is more inhibited, or possibly less supported than L2 in the switching task.

#### Functional Connectivity

**Figure 4:**
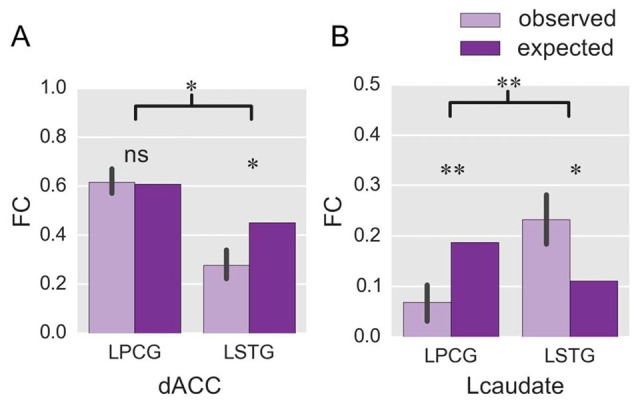
**ROI-wise FC in Experiment 2.** The histograms show the observed FC with the two language-specific regions in the fixed-switch task, and their expected value, for the dACC **(A)** and the Lcaudate **(B)**. The expected FC was defined as the averaged FC across the production tasks with either language in Experiment 1. *p < 0.05; **p < 0.01; ns = not significant. The error bar represents the standard deviation.

We next compared the observed FC in the fixed-switch task to the expected value defined as the averaged FC across the single-language tasks (from Experiment 1). This analysis was designed to investigate how the control regions functionally connect to language-specific regions in a dual-language context. First, the computations of the expected FC value were performed, and the results are shown in Table 1. The following statistical analyses revealed that, compared to its expected value, the observed FC was significantly weaker for the connections of dACC—LSTG (Table 1 and Figure 4A; *p* = 0.017 in *T-test* and *p* = 0.024 in the bootstrap test) and not different for the connection of dACC—LPCG (Table 1 and Figure 4A; *p* = 0.879 in *T-test*; *p* = 0.854 in the bootstrap test). The decrease of the observed FC, relative to its expected value, was more prominent for the connection of dACC—LSTG (Figure 4A; *p* = 0.046 in *T-test* and *p* = 0.044 in the bootstrap test). These results indicate that in the dual-language context (i.e., switching), the dACC modulates the signed language region, but not the spoken language region.

By contrast, compared to its expected value, the observed FC was significantly weaker for the connection of Lcaudate—LPCG (Table 1 and Figure 4B; *p* = 0.008 in *T*-test and *p* = 0.010 in the bootstrap test), and stronger for the connection of Lcaudate—LSTG (Table 1 and Figure 4B; *p* = 0.027 in *T-test* and *p* = 0.030 in the bootstrap test). The differences of FC (observed vs. expected FC) significantly differed between these two connections (Figure 4B; *p* = 0.003 in *T-test* and *p* = 0.008 in the bootstrap test). This finding suggests that the Lcaudate decreases connectivity with the signed language region and increases connectivity with the spoken language region in a dual language context.

#### Brain Activation

**Figure 5:**
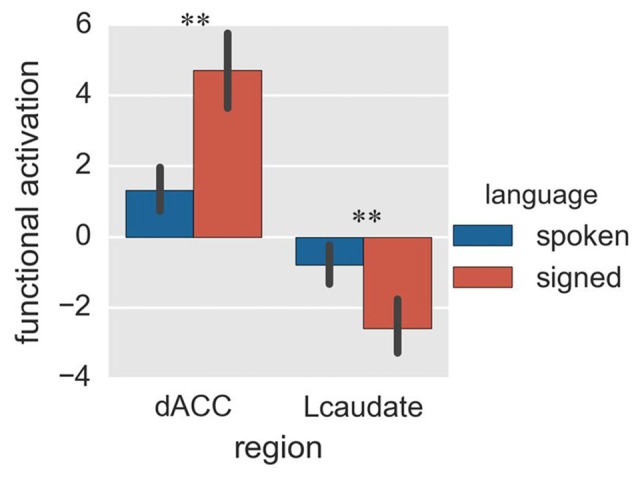
**Activation of the control regions in Experiment 2.** The histogram shows the regional activation of dACC and Lcaudate between signed and spoken languages in the random-switch task. **p < 0.01. The error bar represents the standard deviation.

Finally, we compared activation of the control regions for spoken and signed language production in the random-switch task. The dACC was more activated during signed language production than during spoken language production (Figure 5; *p* = 0.002 in *T*-test and *p* = 0.005 in the bootstrap test), while the Lcaudate was more activated in spoken language than in signed language (Figure 5; *p* = 0.010 in *T-test* and *p* = 0.023 in the bootstrap test). This result was in accord with the pattern of FC differences between the two control regions in terms of their connections with the language-specific regions, suggesting that the dACC exerts control over CSL (L2) and the Lcaudate exerts control over Mandarin (L1) in a dual-language context.

## Discussion

The present study aimed to explore how the dACC and Lcaudate control the language system of bilinguals by examining single- and dual-language contexts. We found that when only one language was used, the dACC mainly controlled the target language in use, while the Lcaudate showed more prominent FC for the less proficient language (CSL). Furthermore, in the language-switching task when both languages were used alternately, the dACC exhibited greater FC with the less proficient language region (the LPCG), while the Lcaudate exhibited greater FC with the L1 region (LSTG). The L1 (Mandarin) region was not strongly connected with the dACC in the language-switching context. These results clarify the distinct roles of dACC and Lcaudate in bilingual language control, and provide direct neural evidence regarding how language control processes adapt to the distinct task demands within different interactional contexts.

For language production in a single-language context, the target language (the language in use) remains unchanged across an entire experimental session. It has been proposed that the role of dACC is to monitor language conflict which arises from the co-activation of the non-target language (Abutalebi et al., 2012; Branzi et al., 2016). If the dACC does monitor language conflict, it should maintain a tight link with the brain regions for both languages, and particularly with regions supporting the non-target language. For example, one might expect that the FC for dACC—LSTG (the region for spoken language) to be equal (or even larger) when signed language is being produced as compared to when spoken language is being produced. However, the results showed that the dACC was more connected with the target language region. There was a significant interaction between connection and language in Experiment 1. The FC of dACC—LSTG was larger in spoken than signed language production, while the FC of dACC—LPCG (the region for signed language) was larger during signed than spoken language production. This result is nicely consistent with the findings from our previous study (Li et al., 2015), in which the FC of dACC—LSTG for the bilingual group was larger than that for monolingual speakers during spoken language naming. The increased connectedness was interpreted as an indicator of more control demand over the target language (L1) for the bilinguals, given that their L1 receives less use compared to monolinguals who do not divide their production across two languages (Gollan et al., 2008; Ivanova and Costa, 2008; Bialystok, 2009; Costa and Sebastián-Gallés, 2014).

Increased FC between the dACC and the target language regions could possibly indicate the control process of monitoring and promoting the activation level of the target language. This idea is consistent with previous proposals that the dACC plays a role in cognitive control by directing attention to task-relevant events (target language processing in our case; Weissman et al., 2005; Orr and Weissman, 2009). Thus, we suggest that the dACC may minimize or resolve language competition by the means of boosting activation of the target language. Such a control process is also implicated in the adaptive control hypothesis proposed by Green and Abutalebi (2013). For example, maintaining the goal of speaking in a target language may involve directing attention to this language and promoting it. However, it is still an open question whether the dACC promotes each response (each trial) in the target language (local control; De Groot and Christoffels, 2006) or promotes the entire language in a sustained way (global control; see below for more discussion of this point). However, the operational definition and the neural substrates underlying local vs. global control are still under dispute (Guo et al., 2011; Branzi et al., 2016).

As for the Lcaudate, we found that it was equally connected with the LPCG (the region for signed language) when producing either CSL or Mandarin within the single-language context. We also found the Lcaudate was more connected with the LSTG (the region for spoken language) when producing CSL than when producing Mandarin. Previous research indicates the Lcaudate plays an important role in the selection of an appropriate response among competitors (Grahn et al., 2008), particularly when control processes have to be recruited (Friederici, 2006). Processing of the less-proficient signed language is less automatic than the dominant spoken language, and may thus engage the control circuit of the Lcaudate to a greater extent. For late bilinguals, their L2 is often acquired by establishing connections to L1, and so they sometimes may retrieve the L2 words via lexical connections with their L1 (Kroll and Stewart, 1994). It is possible that the increased FC with the L1 region (the LSTG) reflects a control process to avoid potential interference with L2 production. Meanwhile, the equally strong connection with the L2 region (the LPCG) for spoken and signed language production may suggest a consistent connection between the Lcaudate and the less-proficient language, regardless of whether it is the target language or not. These findings are consistent with previous studies indicating the Lcaudate is more important for controlling the less proficient language (Tan et al., 2011; Zou et al., 2012b).

In a dual-language context, the neural circuits of language control are engaged in different ways because either language can be the target for production. In Experiment 2, we investigated how the control regions work with each language-specific region under these circumstances. Our results revealed that the dACC modulated signed language production and the Lcaudate modulated spoken language production. This result suggests that control processes might be differently employed in dual-language contexts. To assess this question, we compared the FC in the fixed-switch task with its expected FC value, which was defined as the average FC during signed and spoken processing in the single-language context from Experiment 1. We hypothesized that if the dACC acts on each response (each trial), it could rapidly shift its functional connection with the target regions as the two languages switched. Then for both languages, the observed FC with the language ROIs may be approximately equal to the expected FC value. However, we found the FC of dACC—LSTG (the region for spoken language) was actually weaker than the expected value in the fixed-switch task. The reduced FC of dACC—LSTG suggests that the spoken language is not connected with, or at least is less supported by the dACC in the fixed-switch task. This finding is consistent with our behavioral results showing a larger mixing cost in reaction times for the spoken language than for the signed language (see Figure 3). Similar findings of larger mixing costs for L1 have been obtained in previous psycholinguistic studies (e.g., Meuter and Allport, 1999; Christoffels et al., 2007).

Alternatively, if the dACC only controls the processing of one language, its observed FC would be more prominent with the region for that language compared to the region for the other language. The results revealed that the FC of dACC—LPCG (the region for signed language) was comparable with the expected value, while the FC of dACC—LSTG was smaller, suggesting that the dACC exerted sustained control over the LPCG during the fixed-switch task. In addition, we further found that regional activation of the dACC was greater for signed than spoken language in the random-switch task, supporting the hypothesis that the dACC may control only the signed language in a dual-language context. As argued above (based on the results of Experiment 1), it seems that the function of the dACC is to promote the target language, as evidenced by increased FC with the region for the target language (Li et al., 2015). Thus, the less proficient signed language would receive more support from the dACC than the spoken language. We propose that in the dual-language context, a language control strategy is adopted that aims to achieve a balance between activation levels of the two languages, and that this “language balancing” strategy benefits overall performance in the switching task. One way to achieve such a balance is to inhibit the activation of L1 (Green, 1998), and the another way is to continuously promote the activation of the less proficient L2 (Mayr and Kliegl, 2003; Philipp et al., 2007). The greater FC between the dACC and the neural substrates subserving L2 (signed language here) may be a critical mechanism for the promotion of L2 activation. In short, our results suggest that the dACC exerts control over one of the target languages, specifically, the less proficient one when the languages in use are rapidly changed.

As for the Lcaudate, this region also displayed a distinct pattern of FC in the dual-language context compared to the single-language context. Specifically, compared to the expected value, the FC of Lcaudate—LSTG was increased, while the FC of Lcaudate—LPCG decreased. This result indicated that the Lcaudate was more connected with the more proficient L1. This reverse pattern of connectivity (i.e., to the L1 rather than L2) in the dual-language context appears to contradict previous findings that the Lcaudate mainly controls the less proficient L2 (Tan et al., 2011; Abutalebi et al., 2013). However, this result may also be due to the language balancing strategy adopted in the language-switching situation. Since the L1 may receive less support while the L2 receives sustained support from the dACC, the L1 may become less accessible. The Lcaudate then exerts control over this now less-accessible language. In a single-language context, the less proficient L2 is always the less accessible language, but in a dual-language context, the dominant L1 becomes less accessible. Taken together, it seems the Lcaudate may control the selection of the less accessible language.

The change of accessibility for the two languages of bilinguals has been reported in previous studies (Abutalebi et al., 2009; Hyltenstam et al., 2009). For example, Abutalebi et al. (2009) investigated the language recovery of a bilingual aphasic patient who received language treatment in his L2. They found that as the speech therapy proceeded, his L2 improved and became even more accessible than his L1. The authors further explored the change of FC of the control regions in picture naming tasks with this patient. Most connections within the control regions and the naming network showed larger FC for L2 than L1 naming; however, as the patient’s L2 improved, the connections of the Lcaudate displayed the opposite pattern, i.e., stronger FC for L1 than L2 naming. This change in connectivity is in accord with our finding that in the switching task, the Lcaudate had stronger FC with the L1 (spoken language) which we hypothesize became less accessible with less support from the dACC. In addition, this interpretation was supported by the finding that the Lcaudate was more activated for the spoken language than the signed language condition in the random-switch task.

Our findings support the claim of the adaptive control hypothesis that control processes in bilinguals will change to adapt to different language contexts (Green and Abutalebi, 2013). The findings further provided details regarding how the dACC and Lcaudate work together in controlling a bilingual’s two languages. The results suggest that the role of the dACC is to monitor and modulate the activation level of the target language. In a situation where both languages could alternately be selected as the target language, i.e., in a dual-language context, greater language conflicts are expected. In this case, the less proficient language requires support from the dACC and thus, the dACC may continuously connect with the less proficient target language. We suggest that the role of the Lcaudate is mainly to control the selection of the less accessible language, which is the L2 in a single-language context and the L1 in a dual language context. That is, in a dual-language context, the L1 temporarily becomes less accessible, perhaps because it receives less support from the dACC. In this case, the Lcaudate shows more functional connection with the L1 than the L2. Although the control regions change the pattern of how they interact with the language regions in different language contexts, there are nonetheless some consistent principles. Specifically, the Lcaudate may be always associated with the less accessible language, but which language is less accessible varies in different contexts.

Given that the language-switching tasks in Experiment 2 were more difficult than the tasks in Experiment 1, one may argue that task difficulty would confound the findings of Experiment 2. We had set the duration of each trial in the fixed-switch task to 4 s, slightly longer than that in the single-language tasks (3 s). For the random-switch task, the duration of each trial was even longer. Such a manipulation could allow the participants to make responses at a slow pace, and reduce the difficulty for the language-switching tasks. Moreover, the FC in the fixed-switch tasks actually showed a pattern of varied difference, in comparison with the expected FC values, suggesting that there was no systematic confounding effect of task difficulty. Finally, the consistent findings in both the fixed-switch and random-switch tasks indicate that the different level of response pace, which may imply different task difficulty, did not influence the results. Hence, it seems that task difficulty does not confound the results of the study.

We note that the present study focused on FC without directly assessing the direction of the connectivity between regions, and we also selected a limited number of representative ROIs. Future studies are needed to confirm that the control regions are indeed acting upon the specified language regions (rather than the other way around). Future work is also needed to examine connectivity within the complete language production network. In addition, our experiments were carried out at the word level, and thus could not investigate the processes that occur within an utterance (i.e., dense code-switching). Finally, we recognize that the sample size of this study was relatively small due to the scarcity of bimodal bilinguals, and thus the statistical power was limited.

In summary, for bilingual language control, the dACC monitors and supports the processing of the target language, but not the non-target language. This result is consistent with our previous findings (Li et al., 2015). In addition, the dACC preferentially supports the less proficient L2 if both languages are used alternately in a dual language context. By contrast, the Lcaudate always controls the selection of the less accessible language, which is generally most critical for the less proficient L2. However, if the L1 becomes relatively less accessible as happens in a language-switching situation, then the Lcaudate can also control selection of the L1.

## Author Contributions

LL, KE and GD designed research. LL and XF performed research. LL, XF and GD analyzed data. LL, KE, XF, CL and GD wrote and approved the article.

## Conflict of Interest Statement

The authors declare that the research was conducted in the absence of any commercial or financial relationships that could be construed as a potential conflict of interest.

## Footnotes

^1^The raw data from the current Experiment 1 has been analyzed in a different way and reported in the second experiment in the study by Zou et al. (2012a).
